# Supplementary material for: Short-Term Effects of Cenchrus fungigraminus/Potato or Broad Bean Interplanting on Rhizosphere Soil Fertility, Microbial Diversity, and Greenhouse Gas Sequestration in Southeast China
Source: Microorganisms. 2024 Aug 13;12(8):1665. doi: 10.3390/microorganisms12081665 (PMC11356856; doi:10.3390/microorganisms12081665)
Supplement: Supplementary file 1 [file microorganisms-12-01665-s001.zip › microorganisms-3085893-supplementary.pdf]

Supplementary file

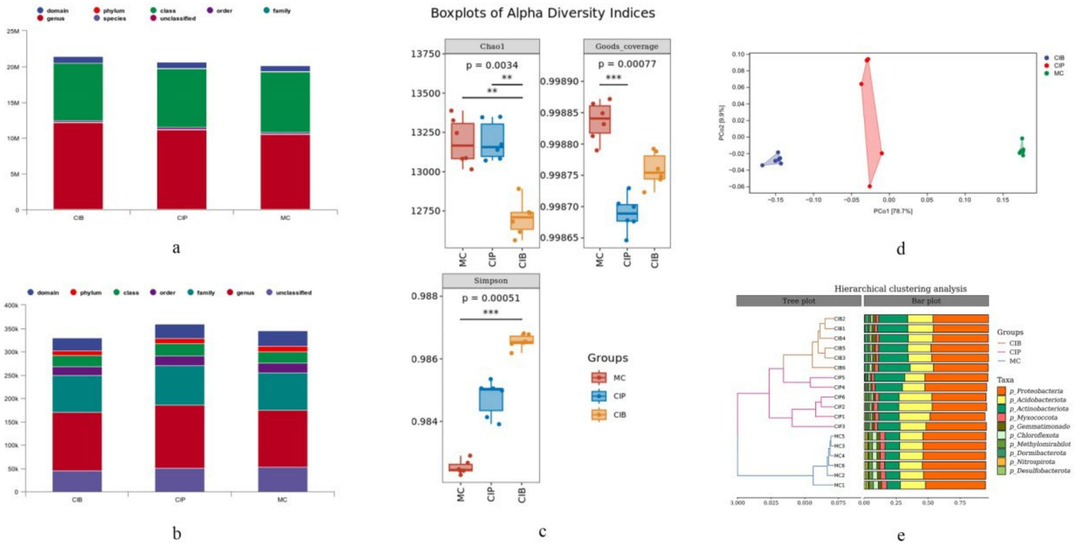

Figure S1. (a) Read sequences of the three groups for species annotation. (b) Species contig abundances in the three groups. (c) Microbial community alpha diversity indexes of the three groups. (d) PCoA of the three groups. (e) Hierarchical clustering analysis at the genus level of the three groups.

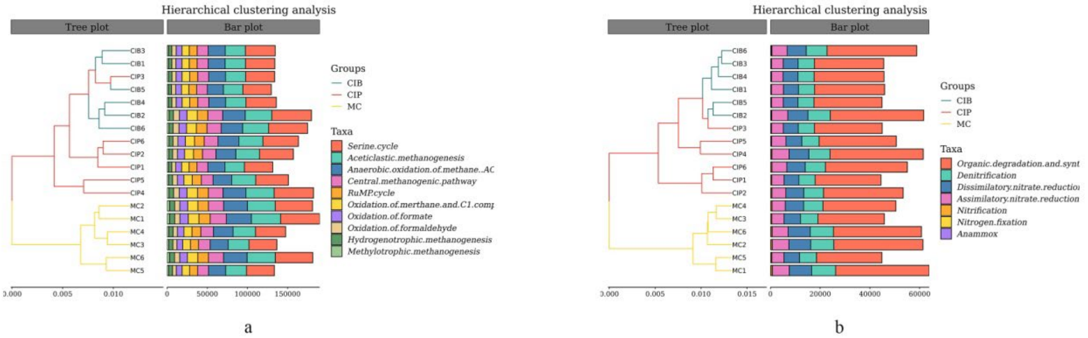

Figure S2. hierarchical clustering analysis of annotated gene functional pathways involved in methane cycle (a) and nitrogen cycle (b).

Table S1. The weather condition in Shunchang County from May 2022 to July 2023.

Year	Month	Temperature (℃)	Average maximum temperature (℃)	Relative humidity （%）	Number of rainy days	Number of hail days	Number of freezing days	Number of snowfall days
2022	May	21.3	25.9	83.4	20	0	0	0
2022	June	25.8	30.1	84.6	20	0	0	0
2022	July	30.7	36.8	68.8	8	0	0	0
2022	August	31.3	37.6	63.1	7	0	0	0
2022	September	28.2	34.1	56.6	0	u0	0	0
2022	October	23.5	29	54.6	1	0	0	0
2022	November	19.7	23.7	82.4	22	0	0	0
2022	December	9.8	13.3	74.8	12	0	0	0
2023	January	10.9	15.4	73.9	9	0	0	0
2023	February	13.6	18.3	72.4	12	0	0	0
2023	March	17.1	22.3	68.2	14	0	0	0

2023	April	20.7	25.4	73.7	20	0	0	0
2023	May	24.3	29.4	73.1	19	0	0	0
2023	June	27.1	32.5	78.4	22	0	0	0
2023	July	30	35	71.2	17	0	0	0

Table S2. Analysis of rhizosphere soil amino sugar composition in all treatments.

**Treatments**	**GalN ($mg \cdot g^{-1}$)**	**ManN ($mg \cdot g^{-1}$)**	**GlcN ($mg \cdot g^{-1}$)**	**Mur ($mg \cdot g^{-1}$)**	**Total ($mg \cdot g^{-1}$)**
OS	0.14 ± 0.04^{d}	0.14 ± 0.02^{c}	0.17 ± 0.03^{d}	1.90 ± 0.35^{b}	2.35 ± 0.11^{c}
MC	0.44 ± 0.05^{b}	0.47 ± 0.07^{b}	0.62 ± 0.05^{b}	2.32 ± 0.17^{b}	3.85 ± 0.09^{b}
CIP	0.22 ± 0.05^{c}	0.29 ± 0.06^{bc}	0.38 ± 0.06^{c}	3.48 ± 0.97^{a}	4.37 ± 1.59^{ab}
CIB	0.64 ± 0.05^{a}	1.12 ± 0.37^{a}	0.88 ± 0.12^{a}	2.31 ± 0.23^{b}	4.95 ± 0.74^{a}

Table S3. Beta diversity index of microbial community among the three treatments.

**Group1**	**Group2**	**Sample size**	**Permutations**	**R**	**p-value**	**q-value**
All	-	18	999	0.8535	0.001	-
CIB	CIP	12	999	0.5556	0.001	0.003
CIB	MC	12	999	1	0.003	0.0045
CIP	MC	12	999	0.7981	0.006	0.006
